# Dynamic profiles and predictive values of some biochemical and haematological quantities in COVID-19 inpatients

**DOI:** 10.11613/BM.2022.010706

**Published:** 2022-02-15

**Authors:** María José Castro-Castro, Laura García-Tejada, Ariadna Arbiol-Roca, Lourdes Sánchez-Navarro, Loreto Rapún-Mas, Isabel Cachon-Suárez, Marta Álvarez-Álvarez, Dolors Dot-Bach, Roser Güell-Miró, Anna Cortés-Bosch de Bassea, Macarena Dastis-Arias, Ana Sancho-Cerro, Noelia Díaz-Troyano, Teresa Escartín-Diez, Diego Muñoz-Provencio, Rosa Navarro-Badal

**Affiliations:** 1 Biochemistry Core of the Clinical Laboratory, Bellvitge University Hospital, Barcelona, Spain; 2 Haematological Core of the Clinical Laboratory, Bellvitge University Hospital, Barcelona, Spain

**Keywords:** COVID-19, pneumonia, prognosis, SARS-CoV-2

## Abstract

**Introduction:**

Severe acute respiratory syndrome coronavirus-2 (SARS-CoV-2) infection in some hospitalized patients has shown some important alterations in laboratory tests. The aim of this study was to establish the most relevant quantities associated with the worst prognosis related to COVID-19.

**Materials and methods:**

This was a descriptive, longitudinal, observational and retrospective study, in a cohort of 845 adult inpatients from Bellvitge University Hospital (L’Hospitalet de Llobregat, Barcelona, Spain). A multivariate regression analysis was carried out in demographic, clinical and laboratory data, comparing survivors (SURV) and non-survivors (no-SURV). A receiver operating characteristic analysis was also carried out to establish the cut-off point for poor prognostic with better specificity and sensibility. Dynamic changes in clinical laboratory measurements were tracked from day 1 to day 28 after the onset of symptoms.

**Results:**

During their hospital stay, 18% of the patients died. Age, kidney disease, creatinine (CREA), lactate-dehydrogenase (LD), C-reactive-protein (CRP) and lymphocyte (LYM) concentration showed the strongest independent associations with the risk of death in the multivariate regression analysis. Established cut-off values for poor prognosis for CREA, LD, CRP and LYM concentrations were 75.0 μmol /L, 320 U/L, 80.9 mg/L and 0.69 x10^9^/L. Dynamic profile of laboratory findings, were in agreement with the consequences of organ damage and tissue destruction.

**Conclusions:**

Age, kidney disease, CREA, LD, CRP and LYM concentrations in COVID-19 patients from the southern region of Catalonia provide important information for their prognosis. Measurement of LD has demonstrated to be very good indicator of poor prognosis at initial evaluation because of its stability over time.

## Introduction

At the end of 2019, a cluster of pneumonia cases of unknown etiology was reported in Wuhan (Hubei Province, China). On January 30th, the World Health Organization (WHO) declared Coronavirus Disease 2019 (COVID-19) as a public health emergency of international concern (*1*, *2*). The next day, the first case in Spain was declared. The virus responsible is a species of SARS-related coronavirus, a novel enveloped RNA virus, which was named severe acute respiratory syndrome coronavirus-2 (SARS-CoV-2) (*1*).

SARS-CoV-2 is transmitted person-to-person through droplets or direct contact, although it began as a zoonotic disease associated with a seafood market. Based on the evidence of rapidly increasing incidence of infections and the possibility of transmission by asymptomatic carriers, SARS-CoV-2 can be transmitted effectively among humans (*3*). In addition, the advancement and convenience of global travel may further enhance its worldwide spread (*4*). Primarily, COVID-19 patients have fever, myalgia or fatigue, and a dry cough at the time of admission. Although most patients have a mild clinical course with broad range symptoms, COVID-19 causes a complicated clinical situation for people with underlying conditions, including diabetes, hypertension, and cardiac, kidney and respiratory diseases, which result in rising rates of hospitalisation and mortality (*5*, *6*). Notably, the highest number of comorbidities has been seen in infected patients admitted to the intensive care unit (ICU), suggesting that chronic diseases are likely to be risk factors for adverse clinical outcomes (*4*).

Since the outbreak of the pandemic, COVID-19 has been an important challenge for hospital care, both due to the significant increase in healthcare pressure and the need to optimise the clinical management of the new disease and its associated pathology, which was unknown at the time.

Beyond the recognised risk factors, several laboratory markers (biochemical, haematological and coagulation quantities) have been identified that indicate the course of COVID-19. The most studied biological quantities in COVID-19 disease are related to the main organs damaged in the infection by this virus: lung, kidney, liver, muscular system, heart. Determining the role of these dynamic blood biochemical changes in the course of the infection is of great importance, and plays an essential role in estimating patients’ diagnosis and prognosis (*2*, *7*). Moreover, identification of laboratory quantities capable of discriminating between cases with a low and high risk of mortality will allow for improved clinical situational awareness.

Most of the studies to date have been conducted with a limited number of cases and only a few biomarkers have been investigated. Therefore, in this retrospective study, we studied changes in laboratory tests in a large group of COVID-19 patients, including more laboratory quantities (*7*).

The aim of this study was to establish the most relevant laboratory quantities associated with the worst prognosis in order to identify early on patients at risk of severe disease progression, and to monitor the dynamic laboratory quantities during hospitalisation.

## Materials and methods

### Subjects/Materials

This was a descriptive, longitudinal, observational and retrospective study that includes a cohort of 845 adult inpatients from Bellvitge University Hospital (L’Hospitalet de Llobregat, Barcelona, Spain). The recruitment period was from March 16^th^ 2020 to April 5^th^, 2020. The follow-up censoring data was June 8^th^ 2020. This period of time includes the majority of patients diagnosed with COVID-19 in the first wave of the COVID-19 pandemic in our hospital.

Our study represents one of the few longitudinal studies on laboratory characteristics in COVID-19 patients with poor and good outcomes. To the best of our knowledge, it is the first study representing the Spanish population.

All consecutive adult patients discharged or deceased after hospital admission with SARS-CoV-2 infection were eligible for inclusion in the study. COVID-19 was diagnosed using a positive result of real-time reverse transcriptase-polymerase chain reaction (RT-PCR) testing of oro/nasopharyngeal swab sample according to the WHO interim guidance.

The inclusion criteria were: a) patients > 18 years, b) confirmed COVID-19 diagnosis, c) hospital discharge or death in hospital. The exclusion criterion was subsequent admission of the same patient.

Personal data relating to patients obtained through the present study were processed in accordance with the Regulation (UE) 2016/679 of the European Parliament on Data Protection. In addition, the study complied with the ethical principles for medical research involving human subjects adopted in the Declaration of Helsinki of the World Medical Association. The Research Ethics Committee of the Bellvitge University Hospital approved this paper for its publication.

### Methods

Demographic, clinical and laboratory data were extracted retrospectively from electronic medical records and the laboratory information. Demographic data collected included age and gender, and the clinical data recorded were ICU admission, diabetes, hypertension, and cardiac, kidney and respiratory diseases. Data on the following laboratory quantities were collected: alanine transaminase (ALT), albumin (Alb), alkaline phosphatase (ALP), aspartate aminotransferase (AST), bilirubin (TBIL), calcium (Ca), creatine kinase (CK), creatinine (CREA), C-reactive protein (CRP), D-dimer (DD), ferritin (FER), gamma-glutamyltransferase (GGT), haemoglobin (Hb), interleukin-6 (IL-6), lactate dehydrogenase (LD), leukocytes (WBC), lymphocytes (LYM), neutrophils (NEU), platelets (Plt), potassium ion (K), procalcitonin (PCT) and troponin T (TnT). Laboratory data chosen depicted the second test result occurring within the 48h from presentation to the Emergency department, coinciding with the routine analytics, which presents a greater number of requested laboratory quantities. Due to the fact that it is a retrospective study, there are no results for all the laboratory parameters in all patients.

To determine the major clinical features that appeared during hospitalization, the dynamic changes in 22 clinical laboratory measurements, including haematological and biochemical measurements, were tracked from day 1 to day 28 after the onset of symptoms, at 3-day intervals. It was decided to pool the results of three days to obtain a greater number of data, since blood tests were not performed on all patients every day.

Venous blood was collected by routine phlebotomy using the BD Vacutainer Serum Separator II Advance Tubes (SST II) (ref. 366468) and the VACUETTE Plasma Lithium Heparin Blood Collection Tube (ref. 454029) for biochemical biomarkers, the VACUETTE TUBE 3.5 ml 9NC Coagulation sodium citrate 3.2% (ref. 474327) for DD, and the BD Vacutainer Plastic K3EDTA Tube 3ml with Lavender Hemogard Closure (ref. 368857) for haematological parameters.

Biochemical biomarkers were measured by spectrophotometric assays using a Cobas e502/e702 (Roche Diagnostics, Basel, Switzerland) and by electrochemiluminiscence assays, using a Cobas e602/e801 (Roche Diagnostics, Basel, Switzerland).

D-dimer was analysed by latex turbidimetric principle using an ACL-TOP 550/750 (Werfen Solutions, Barcelona, Spain).

Cell blood count was analysed by spectrophotometric, impedance and flux citometry using Sysmex XN-10/20 (Sysmex Corporation, Kobe, Japan).

All measurement systems have been correctly checked. Therefore, internal quality control materials have been processed daily, in accordance with the requirements of our laboratory. Quality specifications were met during the period studied.

The clinical laboratory is accredited according to the Internal Standard ISO 15189:2012.

### Statistical analysis

Continuous variables were tested using the Shapiro-Wilk test to check the normality of distribution. Demographic, clinical and laboratory data were compared between survivors and non-survivors using the Chi-squared test for categorical data and the Mann-Whitney U test for continuous data.

To define the independent predictors associated with in-hospital death, a univariable logistic regression model was used. Then, a multivariable regression analysis was fitted. We excluded variables from the multivariable analysis if their univariable analysis was not significant. Some variables were excluded after carrying out a correlation matrix evaluation, which allows to assess the correlation between pairs of variables (Pearson’s correlation coefficient). Those magnitudes with a correlation coefficient > 0.5 were discarded. The receiver operating characteristic (ROC) analysis was carried out to pick an optimum cut-off point in biomarker values that presented significant differences after multivariable analysis (throughout the entire hospital stay).

Results were considered to be statistically significant when P < 0.05. Statistical analyses were performed using the Stata software version 12.0 (College Station, TX: StataCorp LP).

## Results

Altogether 150 (18%) patients died during their hospital stay.

Demographic and clinical data of survivors (SURV) and non-survivors (noSURV) are shown in Table 1.

**Table 1:** Demographic and clinical characteristics grouped according to in-hospital mortality

**Characteristics**	**Total patients** **N = 845**	**Survivors** **N = 695**	**Non-Survivors** **N = 150**	**P**
Age, years (median (min–max))	67 (15-98)	64 (15-98)	75 (44-96)	< 0.001
Sex				
Female, N	340	301	39	< 0.001
Male, N	505	394	111	
**Any comorbidity**				
Hypertension, N	418	319	99	< 0.001
Treatment with ACE-inhibitors, N	183	139	44	0.880
Treatment with angiotensin II receptor antagonists, N	111	88	23	0.391
Diabetes, N	216	165	51	0.009
Chronic obstructive lung disease, N	70	48	22	0.002
Chronic kidney disease, N	112	83	29	0.015
Congestive heart failure, N	79	51	28	< 0.001
Data are reported as the total number of patients with available data (N). P values indicate comparisons between survivors and non-survivors. Results where P < 0.05 were considered statistically significant. ACE-inhibitors - angiotensin-converting-enzyme inhibitors.				

Laboratory data from the second set of tests after hospital admission and their comparison between groups are shown in Table 2. Test for normality using the Shapiro-Wilk test showed that data do not follow a normal distribution. The results of the univariate analysis are shown in Table 3.

**Table 2:** Laboratory findings from the second set of tests after hospital admission, from 845 patients grouped according to in-hospital mortality (survivors/non-survivors)

**Variable (units)**	**Reference range**		**Total patients N = 845**	**Survivors** **N = 695**	**Non-survivors** **N = 150**	**P**
Alanine aminotransferase (U/L)	m	< 41	26 (17–43)	26 (17-44)	27 (17-41)	0.845
	f	< 33				
Albumin (g/L)		35-52	36 (33-38)	36 (33-38)	33 (30-35)	< 0.001
Alkaline phosphatase (U/L)	m	< 129	61 (50-80)	61 (50-76)	65 (54-86)	0.043
	f	< 140				
Aspartate aminotransferase (U/L)	m	< 40	33 (24-49)	30 (23-47)	43 (31-59)	< 0.001
	f	< 32				
Bilirubin, total (µmol/L)		≤ 18	9 (7-13)	7 (6-13)	10 (7-16)	0.018
Calcium (mmol/L)	m	2.20-2.54	2.15 (2.06-2.23)	2.16 (2.07-2.25)	2.09 (2.03-2.18)	< 0.001
	f	2.15-2.51				
C-reactive protein (mg/L)		≤ 5	84 (41-164)	74 (36-140)	176 (96-273)	< 0.001
Creatine kinase (U/L)	m	< 190	77 (44-159)	70 (41-133)	141 (69-269)	< 0.001
	f	< 170				
Creatinine (µmol/L)	m	59-104	76 (62-97)	74 (61-92)	94 (72-131)	< 0.001
	f	45-84				
D- dimer (µg/L)		< 250	313 (250-600)	283 (250-478)	654 (361-1366)	< 0.001
Ferritin (µg/L)		30-400	749 (361-1373)	680 (344-1224)	1218 (714-2004)	< 0.001
Gamma-glutamyltransferase (U/L)	m	≤ 70	44 (24-83)	43 (24-82)	46 (28-90)	0.355
	f	≤ 30				
Haemoglobin (g/L)	m	130-165	130 (119-140)	130 (120-140)	128 (112-139)	0.089
	f	120-147				
Lactate dehydrogenase (U/L)	m	< 225	299 (232-384)	285 (223-355)	425 (348-538)	< 0. 001
	f	< 214				
Leukocytes (× 10^9^/L)		3.9-9.5	6.0 (5.0-8.0)	6.0 (5.0-8.0)	8.0 (5.0-10.0)	< 0.001
Lymphocytes (× 10^9^/L)		1.3-3.4	1.0 (0.7-1.4)	1.1 (0.8-1.4)	0.6 (0.4-0.8)	< 0.001
Neutrophils (× 10^9^/L)		1.5-5.7	4.3 (3.0-6.4)	4.1 (2.9-5.9)	6.4 (4.0-9.1)	< 0.001
Platelets (× 10^9^/L)	m	149-303	214 (159-279)	219 (165-283)	194 (149-257)	0.003
	f	153-368				
Potassium (mmol/L)		3.83-5.10	4.00 (3.68-4.31)	3.98 (3.68-4.29)	4.04 (3.72-4.48)	0.061
Troponin T (ng/L)		≤ 14	11 (7-20)	9 (7-17)	20 (12-36)	< 0.001
Data are reported as median (IQR). P values indicate differences between survivors and non-survivors. Results where P < 0.05 were considered statistically significant. m – male. f – female. IQR – interquartile range.						

**Table 3:** Risk factors for in-hospital mortality (survivors/non-survivors)

	**Univariate OR (95% CI)**	**P**	**Multivariate OR (95% CI)**	**P**
**Demographics and clinical characteristics**				
Female sex (*vs*. male)	0.46 (0.31-0.68)	< 0.001	1.20 (0.64-2.27)	0.568
Age, years*	1.08 (1.06-1.10)	< 0.001	1.11 (1.07-1.14)	< 0.001
**Any comorbidity present (*vs*. not present)**				
Hypertension	2.28 (1.58-3.30)	< 0.001	0.75 (0.39-1.45)	0.397
Treatment with ACE-inhibitors	1.65 (1.11-2.45)	0.014	/	/
Treatment with angiotensin II receptor antagonists	1.31 (0.80-2.15)	0.275	/	/
Diabetes	1.65 (1.13-2.42)	0.010	1.18 (0.61-2.28)	0.624
Chronic obstructive lung disease	2.31 (1.34-3.97)	0.002	1.65 (0.71-3.86)	0.245
Chronic kidney disease	1.76 (1.10-2.81)	0.017	0.32 (0.12-0.84)	0.020
Congestive heart failure	2.89 (1.75-4.76)	< 0.001	1.72 (0.70-4.21)	0.238
Symptom duration before hospital presentation (per day)	0.96 (0.92-1.00)	0.074	/	/
**Laboratory parameters**				
Alanine aminotransferase	0.99 (0.99-1.00)	0.110	/	/
Albumin	0.82 (0.78-0.87)	< 0.001	/	/
Alkaline phosphatase	1.00 (0.99-1.01)	0.191	/	/
Aspartate aminotransferase	1.01 (1.00-1.01)	0.005	/	/
Bilirubin total	1.03 (1.01-1.06)	0.012	/	/
Calcium	0.02 (0-0.11)	< 0.001	/	/
C-reactive protein	1.01 (1.01-1.01)	< 0.001	1.01 (1.00-1.01)	< 0.001
Creatine kinase	1.00 (1.00-1.00)	0.030	/	/
Creatinine	1.00 (1.00-1.01)	< 0.001	1.00 (1.00-1.01)	0.011
D- dimer	1.00 (1.00-1.00)	0.001	1.00 (1.00-1.00)	0.099
Ferritin	1.62 (1.17-2.24)	< 0.001	/	/
Gamma-glutamyltransferase	1.00 (0.99-1.00)	0.674	/	/
Haemoglobin	0.99 (0.98-1.00)	0.027	/	/
Lactate dehydrogenase	1.01 (1.01-1.01)	< 0.001	1.01 (1.00-1.01)	< 0.001
Leukocytes	1.07 (1.03-1.12)	0.001	/	/
Lymphocytes	0.06 (0.03-0.12)	< 0.001	0.22 (0.09-0.51)	< 0.001
Neutrophils	1.16 (1.11-1.23)	< 0.001	/	/
Platelets	0.99 (0.99-0.99)	0.003	/	/
Potassium	1.62 (1.17-2.24)	0.003	/	/
Troponin T	1.00 (1.00-1.00)	0.264	/	/
*Per unit increase. P values indicate differences between survivors and non-survivors, results where P < 0.05 were considered statistically significant. OR - odds ratio. 95%CI - 95% confidence interval. ACE-inhibitors - angiotensin-converting-enzyme inhibitors.				

In the final multivariate model age, kidney disease and CREA, and LD, CRP and LYM concentrations showed the strongest independent associations with the risk of death (Table 3).

**Figure 1:**
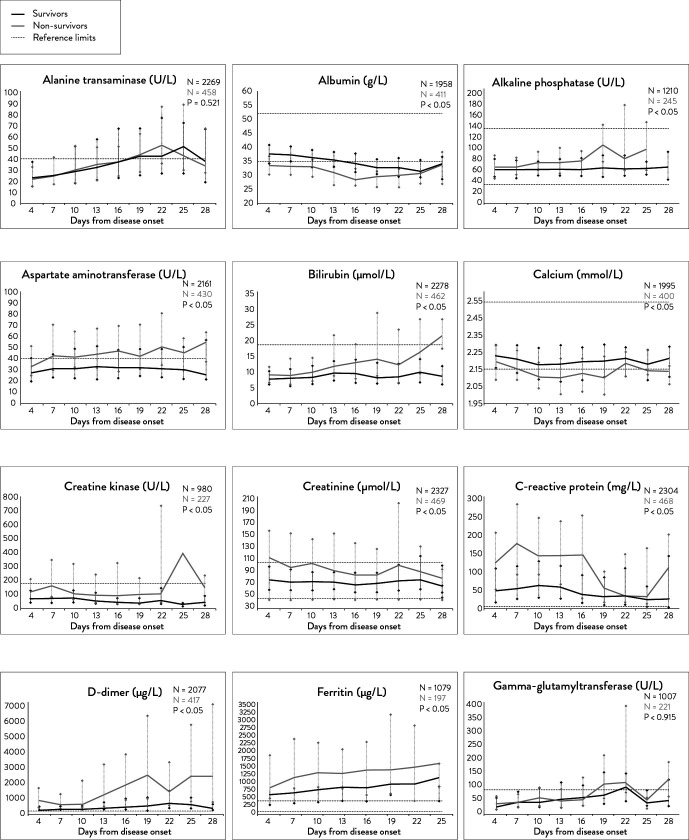
Dynamic changes in laboratory biomarkers during hospitalisation. Dynamic changes at 4-day intervals in alanine transaminase, albumin, alkaline phosphatase, aspartate aminotransferase, bilirubin, calcium, creatine kinase, creatinine, C-reactive protein, D-dimer, ferritin, and gamma glutamyltransferase are shown.

**Figure 2:**
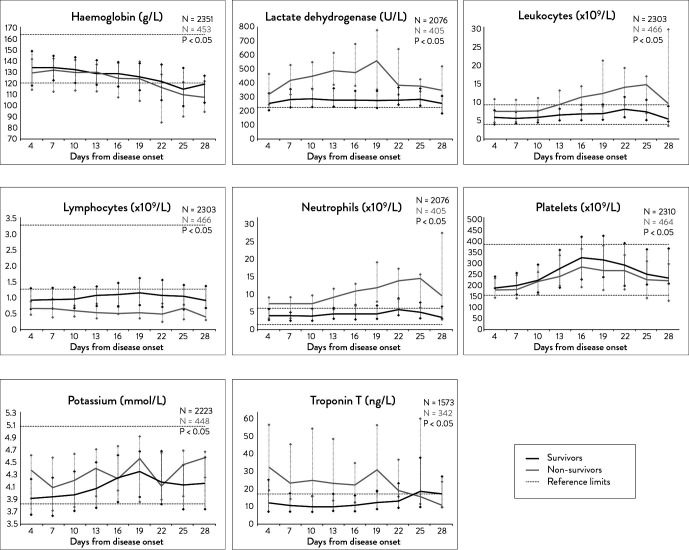
Temporal changes in laboratory biomarkers during hospitalisation. Dynamic changes at 4-day intervals in haemoglobin, lactate dehydrogenase, leukocytes, lymphocytes, neutrophils, platelets, potassium, and troponin T are shown.

Figure 1 and 2 show time series results of biochemical and haematological laboratory measurements.

**Figure 3:**
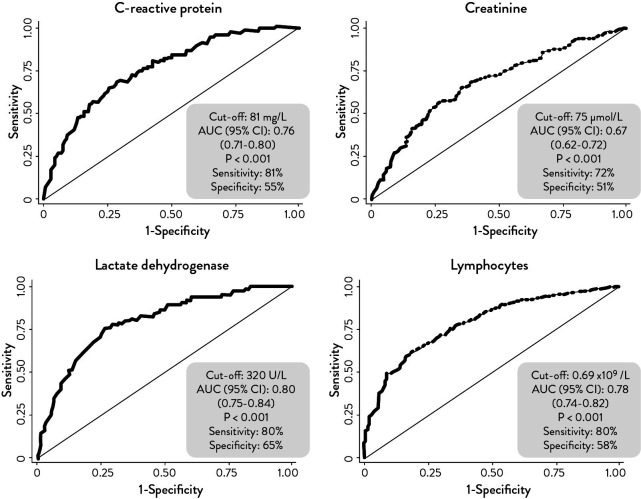
Receiver operating characteristic (ROC) curves and cut-off point in laboratory biomarkers that have presented significant differences after multivariable analysis where P < 0.05 were considered statistically significant. AUC - area under the curve. 95% CI - 95% confidence interval.

Results for ROC curves are shown in Figure 3. Areas under the curve (AUC) of CREA, LYM, CRP and LD concentrations were 0.67 (95% CI 0.62-0.72), 0.782 (95% CI 0.74-0.82), 0.76 (95% CI 0.71-0.80) and 0.80 (95% CI 0.75-0.84), respectively, and cut-off values were 75.0 μmol /L, 0.69 x10^9^/L, 80.9 mg/L and 320 U/L.

## Discussion

The in-hospital mortality in our study was 18%, whereas Berenguer *et al.* reported a mortality rate in other multicenter studies in Spain of up to 28% (*8*). This variability can be attributed to the different criteria for patient admission and different resources available in each centre during the study period. The mortality was higher in men than in women and increased progressively with age as reported by Zhou *et al*., Zhang *et al.* and Berenguer *et al*. (*3*, *4*, *8*).

Procalcitonin, IL-6 and TnT did not show significance in the univariate regression despite the fact that their association with a poor prognosis has been widely described in the literature (*9*-*11*). Limited data available for IL-6 and PCT could affect the obtained results and it would be necessary to include more data in order to determine whether the expected results are obtained or not for this measurement.

Troponin T has been associated with poor prognosis in Spanish population, as stated by García de Guadiana-Romualdo *et al.*, and also foreign populations, as stated by Rath *et al.* and Qin *et al.* (*9*, *12*, *13*). Regarding our data, significant differences were observed between SURV and noSURV groups, but the result of the univariate regression was not significant.

Ferritin did show statistically significant differences in the univariate model. However, it has not been included in the multivariate model due to its correlation with LD, which has been found as a predictor of progression toward severe forms.

Considering the dynamic profile of laboratory findings, most were in agreement with the consequences of organ damage and tissue destruction.

When serial results at 3-day intervals between SURV and noSURV were compared, similar results to laboratory findings from the second set of tests after hospital admission were obtained. The following quantities were significantly increased in noSURV patients: ALP, AST, TBIL, CRP, CK, CREA, FER, LD, K, PCT, TnT, DD, WBC and NEU. However, Alb, Ca, Hb, LYM and Plt concentrations were significantly lower in noSURV patients.

In noSURV group, the measurements which remained stable throughout the studied period were ALB, CK, PCT, K and LD concentrations. The quantities that trended towards an increase were TBIL, ALP, FER, AST, DD, WBC, NEU and Plt concentrations. The quantities that trended toward a decrease over time were LYM, Hb, TnT, Ca and CREA concentrations.

Concerning CREA concentration, despite being significantly higher in noSURV group, it showed a decreasing trend until death occurred. Some studies reported a progressive increase in CREA concentration in noSURV group (*5*, *7*, *14*, *15*). Our data may suggest that a long hospitalisation period and the severity of illness are also associated with the loss of muscle mass, which is directly related to CREA concentration.

Mertoglu *et al.* reported a dynamic profile of higher CK concentration in SURV group than in noSURV (*7*). Considering the ability of the virus to cause tissue destruction or organ damage to the heart and muscle, our results may be consistent from a pathophysiological point of view.

Our study showed Plt concentration with a similar trend and a slight increase over time in both study groups. These results were consistent with Mertoglu *et al.*, who obtained similar results in both groups (*7*). Results of published studies of dynamics of Plt concentration are variable. Ferrari *et al.* and Ding *et al.* showed a marked and increased trend in SURV group over time, and they highlighted this quantity to enhance the assessment of prognosis in hospitalised patients with COVID-19 (*14*, *15*).

According to the measures determined to be significant by the multivariate analysis, a decreasing trend was found in CREA and LYM concentrations in noSURV group. The concentration of CRP, despite having a maintained tendency from day 4 to day 16, showed a decrease up to day 25, with a final rebound in its concentration at day 28. An established cut-off value for each quantity at initial analysis will provide an indicator of prognosis with a certain sensitivity and specificity, but they will vary throughout the period of hospitalisation. In contrast, LD showed a maintained trend in noSURV group throughout the studied period. These results were consistent with Ferrari *et al*., who showed a similar trend in the poor prognosis group from the 5^th^ hospitalization day (*14*). The higher values of LD in noSURV group were in agreement with the extent injuries caused by the virus to lung and kidney. Establishing a cut-off value will be essential for initial analysis and a good indicator of prognosis with sensitivity and specificity values maintained over time.

There were several limitations of this study. First, despite having a great number of cases, this was a single-centre study. Second, the viremia of these patients was unknown. The viral load is a potentially useful marker associated with disease severity and it should be determined in all cases. Concerning laboratory data, as there was no established protocol to request analytics from patients with COVID-19 at the time the study was carried out, there are insufficient data for some measurements (PCT and IL-6) and they may be subject to bias.

This study shows that age, kidney disease, CREA, LD, CRP and LYM concentrations in COVID-19 patients from the southern region of Catalonia provide important information for their prognosis. Some measurements, such as LD, have demonstrated to be very good indicators of poor prognosis at initial evaluation because of their stability over time.
